# Organisational and social work environment factors and occupational balance as predictors of work and life satisfaction among Swedish principals who are also parents to small children

**DOI:** 10.1186/s12889-025-23690-4

**Published:** 2025-07-18

**Authors:** Madeleine Borgh, Roger Persson, Ulf Leo, Carita Håkansson

**Affiliations:** 1 Department of Health Sciences, Lund University, Lund, SE-221 00 Sweden; 2 Department of Psychology, Lund University, Lund, SE-221 00 Sweden; 3 Centre for Principal Development, Umeå University, Umeå, SE-901 87 Sweden; 4 Division of Occupational and Environmental Medicine, Lund University, Medicon Village, Lund, SE-223 81 Sweden

**Keywords:** Managers, Parents, Psychosocial work environment, Work-life balance

## Abstract

**Background:**

In Sweden, managers, individuals working within education, and parents with small children are three groups at high risk for sick leave due to stress-related mental health problems. However, the combined risk of being a parent and manager working within education, i.e., as a principal, on individual work and life satisfaction is not well understood or well-described in the scientific literature. Accordingly, the present study aimed to examine to what extent indicators for occupational balance and organisational and social work environment factors are predictors of work and life satisfaction among Swedish school principals who are also parents to small children.

**Methods:**

A prospective longitudinal study design was used, and data were collected with a one-year interval (T1 and T2) using a web survey. The participants (*n* = 149) had at least one child under 8 years old and answered the survey at T1 and T2. Logistic regression analyses were used to estimate how predictors at T1 determined the reporting of work and life satisfaction at T2.

**Results:**

Supportive organisational structures and few role conflicts at T1 predicted work satisfaction at T2, and supportive organisational structures and a perception of occupational balance at T1 predicted life satisfaction at T2. Adjusted for outcomes at T1, supportive organisational structures and occupational balance still remained predictors.

**Conclusions:**

Supportive organisational structures that clearly define authority and areas of responsibility, and few role conflicts appear to be important for reporting higher work satisfaction among Swedish principals with small children. In addition, high occupational balance and supportive organisational structures that clearly define authority and areas of responsibility also appear to be important for reporting higher life satisfaction. These results suggest that employers of principals with small children may help them by taking action in the above-mentioned areas. The principals themselves could also benefit from being attentive to these issues. Both are important for strengthening public health and preventing sick leave.

## Background

In Sweden, managers, individuals working within education, and parents with small children are three groups at high-risk for sick leave due to stress-related mental health problems [1], and sick leave has been shown to be associated with low work satisfaction [2] and low life satisfaction [3]. Work satisfaction can be seen as a person’s overall experience of their work based on both pros and cons, while life satisfaction encompasses the overall experience of all life domains. Work satisfaction and life satisfaction are important public health constructs [4] and well-being indicators [5]. To strengthen public health and prevent sick leave, it is important to identify predictors of work and life satisfaction. However, the combined risk of being a parent and manger working within education i.e., as a principal, on work and life satisfaction is not well understood or described in the scientific literature. Such knowledge might be useful for deciding how work can be best organised and might suggest specific measures to make it easier for parents to cope with demands both at and outside of work.

In many countries, including Sweden, principals have a comprehensive and complex work role that entails responsibilities and accountability in many dimensions, for example, being responsible for the work environment and for the pedagogical development of both teachers and students. Thus, failure to perform in their work role may spill over and have an impact on teachers’ health and well-being as well as on student learning and thus be detrimental to public health. Previous research among Swedish school principals has shown that 29% reported exhaustion symptoms to a degree that met the criteria for exhaustion disorder on the Karolinska Exhaustion Disorder Scale [6, 7]. Presumably, this high symptom load indicates that school principals have a burdensome middle-management role and that they often must meet conflicting expectations and demands from different stakeholders, for example, the teaching staff, the parents of the students, the state, and the school board [8]. Furthermore, observations of an association between organisational and social work environment factors and perceived work ability [9] and reports of occupational balance [10] suggest that organisational and social work environment factors might negatively influence Swedish principals’ work and life satisfaction.

To maintain health and well-being, it is important to balance activity with recovery [11]. From this perspective, occupational balance is an interesting concept, that describes how well an individual or group of individuals are successful in achieving balance in everyday life. Indeed, occupational balance has been defined as the subjective perception of being satisfied with the amount and variation of different activities in everyday life [12]. In addition, occupational balance entertains the idea that it is necessary to engage in several activities and that it is potentially harmful to focus too much on a single activity (e.g., work) because this may lead to poor recovery and a deselection of activities that provide other meaning [13]. Previous research on occupational balance has, for example, observed a significant association between occupational balance and life satisfaction among adults in Sweden [14, 15]. Coupled parents with high occupational balance have in another study reported both higher work and life satisfaction than couples with low occupational balance [16]. Also, perceiving high occupational balance seems to be an important factor for reporting fewer symptoms of stress among principals in Sweden [10]. Parents of small children have reported difficulties finding time for recovery [17], which is an important aspect of occupational balance [12].

Because the average age of principals in Sweden appears to be on the decline [18], the expectations are that more principals will need to take on the dual responsibility of being a manager and a parent. In Sweden the majority of the parents are living as dual-earner couples [19], and both mothers and fathers need to handle the combined demands from work and life outside work. Swedish parents to children between 3 and 8 years have an increased risk of sick leave due to stress-related mental health problems, explained by the double workload of being a parent and an employee [1]. On the other hand, previous research indicated that being a parent also have positive effects [20], for example by building resilience and support the development of coping strategies to deal with stress in everyday life [21]. Parents in Scandinavian countries report higher levels of happiness compared to non-parents [22], suggesting that being a parent could also be a resource spilling over to work for example when dealing with the demands of being a principal.

While the effects of the combined load from being a principal and a parent, indicating a complexity in juggling the different roles in everyday life, on work and life satisfaction have yet to be examined, there have been studies of work and life satisfaction among principals in different countries. For example, a study of German principals showed that more than a third of the participants reported being dissatisfied or very dissatisfied with their work. Furthermore, the results of this study from Germany and a study from Ireland showed that organisational and social work environment factors seem to contribute significantly to the level of perceived work satisfaction among principals [23, 24]. Organisational and social work environment factors such as decision latitude [23], administrative support, supportive environment among teachers, teachers’ openness for development, and discipline problems [24] were reported to be associated with work satisfaction. Furthermore, a study from the US identified high job demands with unreasonable expectations, managing difficult stakeholders, problematic occupational balance, and lack of support as four themes that influenced work dissatisfaction among school principals [25]. Further, in a study from China work satisfaction was observed to partially mediate a negative correlation between occupational imbalance and the Chinese principals’ work engagement [26]. Another study of Chinese principals showed an association between occupational balance and life satisfaction [27].

Examining work and life satisfaction in principals with small children and its association with the organisational and social work environment as well as occupational balance could give a better understanding of what is important for work and life satisfaction in this target group in order to identify health-promoting factors. Such health-promoting factors might be used to prevent stress-related mental health problems and sick leave. Therefore, the aim of this study was to examine to what extent indicators for occupational balance and organisational and social work environment factors are predictors of work and life satisfaction among Swedish school principals who are also parents to small children.

## Method

A prospective longitudinal study design was used. The data originated from a larger project focusing on Swedish principals’ health and work environment [7]. Data were collected via a web survey that was administered twice with a one-year interval, specifically in 2018 (T1) and 2019 (T2).

### Participants

Principals and assistant principals from pre-school, compulsory school, upper secondary school, and adult education in Sweden were contacted by email. Email addresses were obtained with permission from the Swedish National Agency for Education, and entailed principals who during the period 2008 to 2017 had participated in a principal training programme.

**Fig. 1:**
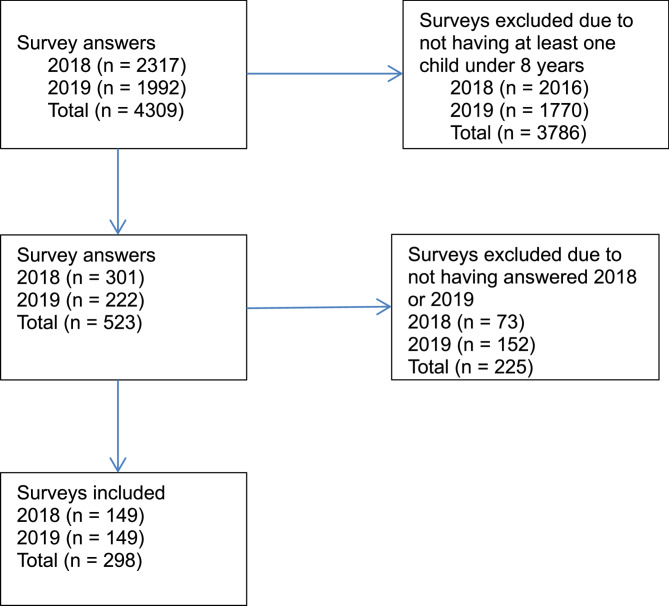
Flow chart of the study sample

Specifically, at T1, 9900 school principals were invited and 4640 individuals either actively declined (*n* = 2007) or accepted (*n* = 2633) participation (i.e., a 47% response rate). Of the individuals who accepted, 2317 individuals completed the survey (i.e., a 23% response rate in relation to all invited and 50% in relation to those who actively responded to the e-mail invitation). At T2, in 2019, all but one retiree among the responders from T1 were reinvited as were all who did not respond to the first invitation at T1. Of the non-responders at T1, 1506 (i.e., 29%) broke silence at T2 and made an active decision to decline (*n* = 882) or accept (*n* = 624) participation, resulting in 464 new individuals who completed the survey at T2 (*n* = 1992), giving a total of 4309 responses. Of the responders at T1 1528 completed the survey at T2 (i.e., 66% of the responders at T1). From this pool of participants, we identified 149 principals having at least one child under 8 years of age at T1 who also answered the survey at T2. For more detailed information see Fig. 1.

### Outcomes

#### Life satisfaction and work satisfaction at T2

The outcome variables were measured using two questions from the Life Satisfaction Questionnaire (LISAT), namely “How satisfied are you with your work?” and “How satisfied are you with your life in general?” [28, 29]. Possible response alternatives were “very dissatisfied” [1], “dissatisfied” [2], “pretty dissatisfied” [3], “pretty satisfied” [4], “satisfied” [5], and “very satisfied” [6]. Higher scores indicated higher levels of life satisfaction and work satisfaction. The LISAT has been found to be valid for the population at large [28, 29], and for this study both work and life satisfaction were dichotomised based on positive and negative answers.

### Independent variables at T1

#### Organisational and social work environment factors

An abbreviated version of the Gothenburg Manager Stress Inventory (GMSI) [30], the GMSI-mini, was used to measure demanding and supportive organisational and social work environment factors. Demanding factors included resource imbalance, organisational governance shortcomings, role conflicts, role demands, group dynamic problems, employee problems, having a buffer function, and having a container function. The supportive factors included supportive management, collaboration with employees, supportive colleagues at the leadership level, supportive organisational structures, and a supportive private life. Response alternatives for supportive factors were “applies very poorly” [1], “applies poorly” [2], “applies to some extent” [3], “applies well” [4], and “applies very well” [5]. For demanding factors, possible responses were “never/almost never” [1], “rarely” [2], “sometimes” [3], “often” [4], and “always/almost always” [5]. A confirmatory factor analysis previously confirmed a good fit between the latent GMSI-Mini areas/dimensions and the observed data in a larger sample of principals [9]. For the present study, the mean scores for each GMSI variable were dichotomised based on the median.

#### Occupational balance

Occupational balance was measured with the Occupational Balance Questionnaire (OBQ11) [31], which consists of 11 statements with the four response alternatives of “don’t agree” [0], “somewhat agree” [1], “agree” [2], and “totally agree” [3]. The sum of responses from all statements is calculated (ranging from 0 to 33) to show the level of occupational balance, with higher scores indicating a higher level of occupational balance. The OBQ11 has shown good validity and reliability [31] as well as good test-retest reliability [32]. For the present study, total sum score of the OBQ11 was dichotomised based on the median [9], into “higher occupational balance (10–33)” vs. “lower occupational balance (0–9)”.

### Covariates at T1

Potential covariates at T1 included in the present study were gender, partner, number of children < 8 years, amount of physical activity, satisfaction with amount of sleep, support in private life, overtime, and numbers of subordinated, and years of experience as a principal.

Physical activity was measured with one question ‘How physically active have you been the last three months’ and dichotomised into mainly sedentary vs. physically active (light/moderate/heavy) [33]. Sufficient sleep had two response alternatives yes vs. no. Response alternatives for support in private life were “Yes, very much” [1], “yes, pretty much” [2], “yes, some” [3], and “No” [4]. Overtime had the eight response alternatives of “non-regulated working hours” [1], “never” [2], “a few times per year” [3], “a maximum of once per month” [4], “a few times per month” [5], “once a week” [6], “a few times a week” [7], and “every day” [8]. Overtime was grouped into “non-regulated working hours”, “never or only a few times a year”, “a few times per week”, and “every day”.

### Statistical analysis

The covariates were analysed for multicollinearity using Spearman’s or Pearson’s correlations. A correlation of < 0.8 was used as the threshold for including covariates, and no covariates were excluded based on this. Next, due to the small sample size, using a *p*-value < 0.1 as criteria for selection, bivariate analyses between the covariates and each dependent variable were performed to decrease the number of variables. To examine possible predictors of work satisfaction and life satisfaction stepwise binary logistic regressions were performed. In the first step the GMSI variables were added. In the second step, the OBQ11 variable was included in the analysis. In step three, the covariates that had a *p*-value < 0.1 in the bivariate analysis were added. This involved adjusting for physical activity for work satisfaction, whereas life satisfaction was adjusted for private social support, satisfaction with amount of sleep, and overtime. In the last step, the analysis was adjusted for either work satisfaction or life satisfaction at T1. A *p*-value 0.05 was considered significant. All statistical analyses were performed in SPSS, version 28.

## Results

Demographic data is presented in Table 1. In the present study, more woman than men participated (57% and 43%, respectively). It was most common to live together with a partner (93%), have one child under 8 years (60%), and be 35 to 45 years of age. The median age was 40 years (30-55years). A great majority reported being physically active, and a majority reported insufficient sleep.

**Table 1:** Descriptive characteristics of the participants at baseline (*n* = 149)

	*n* (%)
Gender	
Women	85 (57)
Men	64 (43)
Partner	
Yes	139 (93)
No	10 (3)
Number of children < 8 years	
1	90 (60)
2	52 (35)
3 or more	7 (5)
Amount of physical activity	
Mainly sedentary	20 (13)
Some physical activity	61 (41)
Moderate physical activity	50 (34)
Intense physical activity	18 (12)
Sufficient sleep	
Yes	60 (40)
No	89 (60)
Support in private life	
Yes, much	41 (27)
Yes, pretty much	56 (38)
Yes, some	40 (27)
No	12 (8)
Overtime	
Non regulated working hours	10 (7)
Never or a few times a year	26 (16)
A few times per week	90 (60)
Every day	23 (16)
Numbers of subordinated	
<20	45 (30)
21-30	42 (28)
31-40	35 (24)
41-120	27 (18)
Years of work experience as a principal	
<5 years	91 (61)
5–10 years	43 (29)
>10 years	15 (10)
Demanding organisational and social work environment (scale 1-5^a^)	Mean (SD)
Resource imbalance	3.4 (1.0)
Organisational governance shortcoming	2.4 (0.9)
Role conflicts	3.6 (0.9)
Role demands	3.0 (0.8)
Group dynamics	2.3 (0.8)
Buffer function	2.7 (1.0)
Co-workers	3.0 (0.8)
Container function	3.4 (0.9)
Supportive organisational and social work environment (Scale 1-5^b^)	Mean (SD)
Supportive management	3.3 (1.1)
Cooperating with co-workers	4.1 (0.7)
Supportive managers and colleagues	3.8 (1.2)
Supportive private life	3.5 (1.1)
Supportive organisational structures	3.6 (1.1)
Occupational balance	
Yes/Higher	78 (52)
No/Lower	71 (48)

^a^Higher scores on the scale indicate less demanding factors

^b^Higher scores on the scale indicate more supportive factors

The median occupational balance score was 9 (Q1 = 5, Q3 = 15). The median split generated slightly more principals in the group who perceived higher occupational balance (52%).

A majority of the participants had less than five years of work experience as principals, and a majority reported working overtime several times every week. Among organisational and social work factors, resource imbalance, role conflicts, and container function were the most demanding for the principals. The strongest supportive organisational and social work factor was cooperation with co-workers (Table 1). The participants’ scores across the two outcome variables are described in Table 2.

**Table 2:** Distribution of work and life satisfaction scores 2018 (T1) and 2019 (T2)

	Work Satisfaction		Life satisfaction	
	T1	T2	T1	T2
	*n* (%)	*n* (%)	*n* (%)	*n* (%)
Very dissatisfied	2 (1)	2 (1)	3 (2)	3 (2)
Dissatisfied	8 (5)	7 (5)	4 (3)	3 (2)
Pretty dissatisfied	18 (12)	16 (11)	11 (7)	12 (8)
Pretty satisfied	46 (31)	61 (41)	43 (29)	43 (29)
Satisfied	53 (36)	47 (31)	68 (38)	68 (46)
Very Satisfied	22 (15)	16 (11)	31 (21)	20 (13)

### Bivariate associations between potential covariates and work and life satisfaction

Bivariate associations with a *p*-value < 0.1 were found between physical activity and work satisfaction as well as between overtime, support in private life, sufficient sleep and life satisfaction (Table 3).

**Table 3:** Bivariate associations between potential covariates and work and life satisfaction

	Work satisfaction		Life satisfaction	
	OR (95% CI)	*p*-value	OR (95% CI)	*p*-value
Gender	0.78 (0.33–1.85)	0.577	1.21 (0.44–3.32)	0.711
Overtime	0.70 (0.39–1.27)	0.242	**0.47 (0.22–1.02)**	**0.056**
Years of experience as a principal	1.26 (0.80–1.98)	0.315	0.95 (0.58–1.57)	0.850
Number of subordinates	0.94 (0.63–1.39)	0.744	0.82 (0.52–1.29)	0.395
Partner	0.79 (0.16–3.98)	0.778	1.25 (0.15–10.53)	0.835
Number of children < 8 years	0.79 (0.40–1.56)	0.505	0.81 (0.37–1.76)	0.592
Physically active	**1.60 (0.95–2.69)**	**0.078**	1.00 (0.57–1.76)	0.994
Support in private life	0.70 (0.45–1.13)	0.146	**0.39 (0.22–0.70)**	**0.002**
Satisfaction with amount of sleep	1.92 (0.75–4.92)	0.175	**6.36 (1.40–28.80)**	**0.016**

The figures in bold have an association with work or lig´fe satisfaction *p* < 0.1

## Work satisfaction

Role conflict was the only one out of eight demanding social and organisational work factors that showed a significant association in the first and second step of the analysis and when adjusted for physical activity. The likelihood for principals with small children to experience high work satisfaction decreased by 90% if role conflicts were reported, but lost significance when adjusted for work satisfaction scores at T1. In contrast, supportive organisational structures showed significant associations with work satisfaction across all four steps and remained significant after adjusting for physical activity, and work satisfaction scores at T1. Principals with small children were almost six times more likely to experience work satisfaction when reporting supportive organisational structures. The results showed no association between occupational balance and work satisfaction (Table 4).

**Table 4:** Organisational and social work factors and occupational balance at T1 as predictors of work satisfaction at T2

	Step 1 OR (95% CI) *p*-value	Step 2 OR (95% CI) *p*-value	Step 3^a^ OR (95% CI) *p*-value	Step 4 OR (95%CI) *p*-value
*Demanding social and organisational work factors*				
Resource imbalance	0.87 (0.15-4.95) 0.867	0.89 (0.15–5.31) 0.895	0.85 (0.14–5.11) 0.855	0.94 (0.15-5.88) 0.945
Organisational governance shortcomings	1.11 (0.23-5.36) 0.899	1.14 (0.23–5.70) 0.876	1.28 (0.24–6.69) 0.773	1.10 (0.19-6.26) 0.913
Role conflicts	**0.06 (0.07-5.29) 0.011**	**0.10 (0.01–0.91) 0.041**	**0.10 (0.01–0.89) 0.038**	0.11 (0.01-1.05) 0.055
Role demands	2.45 (0.31-19.64) 0.399	2.90 (0.31–27.17) 0.352	3.93 (0.37–41.37) 0.254	3.78 (0.33-43.0) 0.284
Group dynamic problems	1.51 (0.22-0.64) 0.678	1.37 (0.18–10.76) 0.763	0.88 (0.10–8.04) 0.910	1.14 (0.11-11.47) 0.911
Buffer function	0.48 (0.11-2.09) 0.329	0.48 (0.11–2.17) 0.339	0.57 (0.12–2.80) 0.486	0.53 (0.11-2.59) 0.432
Employee problems	1.18 (0.18-7.97) 0.866	1.04 (0.15–7.42) 0.969	1.36 (0.18–10.49) 0.766	1.28 (0.17-9.80) 0.815
Container function	1.25 (0.21-7.62) 0.809	1.26 (0.21–7.58) 0.804	0.99 (0.16–6.30) 0.989	0.95 (0.14-6.42) 0.958
*Supporting social and organisational factors*				
Supportive management	1.92 (0.37-9.93) 0.436	1.92 (0.36–10.37) 0.447	2.05 (0.37–11.43) 0.415	1.55 (0.25-9.61) 0.639
Collaboration with employees	1.17 (0.25-5.49) 0.840	1.30 (0.25–6.67) 0.754	1.15 (0.21–6.15) 0.874	1.29 (0.23-7.10) 0.774
Supportive colleagues at the leadership level	2.50 (0.52-11.98) 0.251	2.47 (0.48–12.77) 0.282	2.99 (0.55–16.32) 0.206	2.68 (0.48-14.93) 0.262
Supportive organisational structures	**4.90 (1.11-21.58) **0.036	**5.96 (1.26–28.16) **0.024	**5.57 (1.18–26.24) **0.030	**5.09 (1.07-24.28) **0.041
Supportive private life	0.30 (0.06-1.39) 0.123	0.24 (0.05–1.17) 0.078	0.21 (0.04–1.05) 0.058	0.23 (0.05-1.18) 0.079
High occupational balance vs. low		3.12 (0.65-15.06) 0.323	2.57 (0.51-13.02) 0.254	2.32 (0.44-12.10) 0.319
Work satisfaction at T1				2.32 (0.15-75.35) 0.442

^a^Adjusted for physical activity

Figures in bold are statically significant

## Life satisfaction

No significant associations were found for demanding social and organisational work factors. In fact of all GMSI variables, only supportive organisational structure showed a significant association with life satisfaction in step 3 (i.e. when adjusted for support in private life, satisfaction with sleep and overtime) and step 4 (i.e. after being adjusted for life satisfaction scores at T1). Principals with small children who reported supportive organisational structures were 6.5 times more likely to experience life satisfaction. The second step of the analysis showed a significant positive association between occupational balance and life satisfaction, and this remained significant in step 3 (i.e. when adjusted for support in private life, satisfaction with sleep, and overtime) and in step 4 (i.e. after being adjusted for life satisfaction scores at T1). Principals with small children were 5 to 7 times more likely to report life satisfaction when also experiencing higher occupational balance. When adjusted for life satisfaction at T1, high life satisfaction at T1 also became a predictor of high life satisfaction at T2 (Table 5).

**Table 5:** Organisational and social work factors and occupational balance at T1 as predictors of life satisfaction at T2

	Step 1 OR (95% CI) *p*-value	Step 2 OR (95% CI) *p*-value	Step 3^a^ OR (95% CI) *p*-value	Step 4OR (95% CI)
*Demanding social and organisational work factors*				
Resource imbalance	0.83 (0.20-3.40) 0.798	0.89 (0.20-3.94) 0.876	0.75 (0.16-3.60) 0.717	0.68 (0.13-3.67) 0.650
Organisational governance shortcoming	2.26 (0.58-8.79) 0.241	1.96 (0.49-7.81) 0.343	1.44 (0.31-6.66) 0.639	1.45 (0.28-7.59) 0.663
Role conflicts	0.32 (0.06-1.90) 0.212	0.60 (0.09-4.25) 0.610	0.23 (0.02-2.22) 0.203	0.43 (0.04-4.88) 0.493
Role demands	1.70 (0.27-10.74) 0.574	1.85 (0.25-13.91) 0.551	2.98 (0.34-26.02) 0.323	3.83 (0.37-4.88) 0.493
Group dynamic problems	0.65 (0.13-3.14) 0.589	0.55 (0.10-2.99) 0.489	0.77 (0.13-4.60) 0.774	0.68 (0.10-4.73) 0.696
Buffer function	0.51 (0.14-1.86) 0.310	0.53 (0.14-2.03) 0.350	1.16 (0.21-6.53) 0.863	1.87 (0.30-11.76) 0.504
Employee problems	0.74 (0.17-3.14) 0.679	0.67 (0.15-3.10) 0.609	0.50 (0.10-2.60) 0.409	0.56 (0.10-3.25) 0.521
Container function	1.78 (0.47-6.79) 0.397	1.73 (0.43-6.98) 0.443	1.74 (0.38-8.06) 0.479	0.83 (0.15-4.60) 0.826
*Supporting social and organisational factors*				
Supportive management	1.30 (0.31-5.41) 0.715	1.17 (0.26-5.16) 0.839	1.37 (0.29-6.49) 0.694	1.13 (0.21-5.10) 0.885
Collaboration with employees	0.53 (0.15-1.88) 0.324	0.51 (0.13-2.03) 0.343	0.39 (0.09-1.68) 0.204	0.54 (0.11-2.75) 0.455
Supportive colleagues at the leadership level	1.49 (0.41-5.41) 0.544	1.45 (0.36-5.83) 0.601	1.94 (0.44-8.66) 0.384	1.93 (0.36-10.48) 0.444
Supportive organisational structures	2.92 (0.78-11.09) 0.115	3.72 (0.87-15.91) 0.077	**6.47 (1.19-35.12) **0.031	**9.10 (1.35-61.55) **0.024
Supportive private life	1.05 (0.33-3.37) 0.936	0.67 (0.18-2.50) 0.554	0.52 (0.12-2.24) 0.381	0.34 (0.07-1.76) 0.197
*High occupational balance vs. low*		**5.05 (1.35-18.90) **0.016	**6.71 (1.33-34.03) **0.021	**6.60 (1.26-34.45) **0.025
*Life satisfaction at T1*				**7.90 (1.50-41.50) **0.015

^a^Adjusted for private social support, satisfaction with sleep and overtime

Figures in bold are statically significant

## Discussion

The present study examined a large set of potential predictors of work and life satisfaction among Swedish principals who were also parents to small children (i.e., having at least one child below eight years old). The results showed that supportive organisational structures and few role conflicts at T1 predicted the reporting of work satisfaction at T2, although role conflicts lost significance (*p* = 0.055) when adjusting for work satisfaction scores at T1 in the final step. In addition, perceiving occupational balance and the presence of supportive organisational structures at work predicted life satisfaction at T2, even after adjusting for work satisfaction scores at T1.

Noticeably, among the principals with small children investigated here, role conflicts (i.e., conflicts between educational development, administration, and contact with employees) decreased the perception of work satisfaction. Albeit the role conflicts lost significance in the final step, the overall pattern of the current estimates of associations between role conflict and work satisfaction seems to align with another study of nurses and physicians in which it was reported that role conflict was one of the most important predictors of work satisfaction [34]. It is plausible that the principals with small children had the same role conflicts as other principals, that is, to be a good leader, administrator, etc. However, the added role as a parent, with all the ensuing practical and emotional implications, may lead to feelings of inadequacy and a high workload. Indeed, a meta-analysis showed a positive association between role conflicts and workload [35]. That the majority of the participants in the present study also worked overtime every day or a few times per week can also be interpreted as a sign of a high workload that to some extent may be separate from the workload caused by role conflicts. Thus, role conflicts and workload may alone or in combination create job requirements for principals with small children that exceed their resources on more than a temporary basis. This may lead to various stress reactions, and it is known that stress is negatively related to work satisfaction [36].

Supportive organisational structures (i.e., clearly defined authority in one’s work and areas of responsibility) at T1 were important predictors for both work and life satisfaction at T2. The variable of supportive organisational structures covers the conditions that should be included in any job description, that is, a specification of what the employee should do, the responsibilities that need to be undertaken, and what the organisation expects from the employee [37]. Accordingly, this result aligns well with the results of previous research showing that employees tend to be dissatisfied with their work when they do not have a clear job description, and the more employees perform duties outside their job description the lower their work satisfaction [38]. That said, beyond the local job description the responsibilities for principals in Sweden are also regulated in educational laws and curricula and via municipal delegation arrangements. While regulations may be helpful, having the principals’ authority and responsibilities regulated in so many places, and from different stakeholders, can of course lead to role conflicts and contradicting demands [8]. Accordingly, it seems warranted that principals should have access to support from their managers when needed. Support is key for handling conflicting demands, and support from managers has been shown to be important for work satisfaction [39].

Both role conflicts and lack of a clear job description and authority may lead to the principals working too much at the expense of life outside work. If work dominates life, it is hard to perceive occupational balance, which was the strongest predictor of life satisfaction. Interestingly, this result is well aligned with the results of a previous study that showed that conflicts between work and family had a negative relationship with life satisfaction [40]. Further the study by Goh et al. [40] also showed that a high workload (paid work) increases the conflict between work and family, which in turn decreases life satisfaction.

Occupational balance means the perception of having the right amount of and the right variation between paid and unpaid work and recovery [12]. This is not the least important for parents with small children because the children depend on their parents for help and support. In the present study the studied parents with small children had a lower occupational balance (Mdn = 9) compared to other studied groups as for example Swedish principles in general, who had a median of 11 [10] and parents with other employment, who had a median of 11 [41]. It seems that the mean age of principals is decreasing [18], and thus the probability that they have small children is increasing. In order for them to experience work and life satisfaction, it is therefore important that their employers work to improve the organisational and social work environment. That is to say, to make sure that principals have a clear job description, clear authority, and a reasonable workload. All these may also influence their perception of occupational balance, which has been shown to predict health [15, 42], and occupational imbalance predicts stress-related mental health problems [43].

### Strengths and limitations

The present study has both strengths and limitations. A strength is that we were able to identify a relatively large group of parents who also were managers within a specific occupational group (*n* = 149). Beyond our interest in this occupational group, the choice to study one occupational group increases the internal validity of the observed association between the potential predictor variables and work and life satisfaction because any potential occupation-specific confounding is likely limited or absent. However, a limitation is that studying one specific group of managers restrict the generalisability of the results. Hence, further studies that include mangers with small children from other professions are needed to help determine the extent to which it is possible to generalise the results. A second limitation, although several variables related to work and life satisfaction were included in the regression models, there may be additional factors that promote good work and life satisfaction. Thus, the results are not exhaustive, and more research containing other variables seem warranted. Thirdly, it should also be noted that some of the confidence intervals in the logistic regression analyses were rather wide, which means that the estimate is imprecise, and that appropriate caution should be exerted when interpreting the findings. Fourth, since there was a year between the data collection occasions (i.e., T1 and T2), it cannot be excluded that changes in unmeasured factors could have exerted an intermittent influence on the outcomes at T2 (e.g. changes that imply problems at work, in the marriage, or even parental problems). Fifth, while a longitudinal design typically provide a stronger argument for causality than a cross-sectional analysis, and a strength of the present study, it cannot be excluded that an individual’s current life and work satisfaction may influence how this individual report in, for example, GMSI or OBQ (i.e., reversed causality). However, to lessen the risk of reverse causality, we also performed analyses in which we adjusted the outcome scores at T2 for their T1 scores, respectively, thus focusing on estimating the change from T1 to T2 on the outcome variable. 

### Conclusion and further research

Supportive organisational structures that clearly define authority and areas of responsibility appear to be important for reporting higher work satisfaction among Swedish principals with small children. Also experiencing few role conflicts seem to matter for experiencing work satisfaction. In addition, high occupational balance and supportive organisational structures that clearly define authority and areas of responsibility appear important for reporting higher life satisfaction. These results suggest that employers of principals with small children in Sweden may help them by taking actions in the above-mentioned areas. The principals themselves could also benefit from being attentive to these issues. Both are important to make it easier for parents to cope with the demands both at work and outside work and for strengthening public health and preventing sick leave.

In the present study all principals with at least one child below the age of eight years were included. In further studies, it would be of interest to identify differences in work and life satisfaction depending on which school level the principals work. It would also be interesting to interview principals about what they perceive affects their work and life satisfaction.

## Data Availability

The datasets generated and analysed during the current study are not publicly available because the ethical approval by the Regional Ethical Review Board specifies that crude data must not be published on the Internet. The dataset used and analysed during the current study is available from the first author (MB) on reasonable request.
